# Moral-psychological mechanisms of rebound effects from a consumer-centered perspective: A conceptualization and research directions

**DOI:** 10.3389/fpsyg.2022.886384

**Published:** 2022-08-05

**Authors:** Hanna Reimers, Wassili Lasarov, Stefan Hoffmann

**Affiliations:** Department of Marketing, Institute of Business Administration, Kiel University, Kiel, Germany

**Keywords:** moral licensing, rebound effects, categorization, behavioral spillovers, consumption context

## Abstract

Rebound effects on the consumer level occur when consumers’ realized greenhouse gas emission savings caused by behaviors that might be beneficial to the environment are lower than their potential greenhouse gas emission savings because the savings are offset by behavioral adjustments. While previous literature mainly studied the economic mechanisms of such rebound effects, research has largely neglected the moral-psychological mechanisms. A comprehensive conceptualization of rebound effects on the consumer level can help fill this void and stimulate more empirical research in this relevant area. To this end, the paper introduces three focal dimensions of rebound effects on the consumer level: mechanism of rebound effects, product category, and consumption context. Based on this conceptualization, and integrating assumptions from the theory of moral licensing, the theory of categorization, and the construal level theory, this paper further refines the conceptualization of the moral component as an explanatory factor for rebound effects and highlights that the moral-psychological mechanisms of indirect rebound effects (i.e., rebound effects that occur across different product categories or consumption contexts) are more complex and diverse than the economic mechanisms. The paper outlines promising directions for future studies considering the different quantification and characteristics of economic and moral currencies that explain rebound effects on the consumer level and the strategic categorization of products and consumption contexts.

## Introduction

*Imagine the company iReliefs hires two sales representatives, Oeconomica and Moralis, for organic ready-to-eat meals. The company employs Oeconomica due to her experience in her previous job as a financial controller, while it employs Moralis because her sustainability attitudes match the company’s philosophy. From their first salary, both decide to buy an e-bike for private purposes to replace car rides by e-bike rides. Oeconomica calculates her savings over time through switching from car to e-bike, and spends the gained financial resources on foreign travel. Moralis concludes that switching to the e-bike is a moral deed. Since she reduced her carbon footprint, she feels liberated to act less sustainably in other consumption areas. Subsequently, Moralis will choose the meat dish over the vegetable dish in the canteen at work more often. Whereas replacing their cars with e-bikes activates different mechanisms for Moralis and Oeconomica, the consequences are similar: Greenhouse gas emission-intensive behavioral responses attenuate the potential greenhouse gas emission savings, i.e., a rebound effect on the consumer level.*

Rebound effects refer to the failed realization of potential greenhouse gas emission savings. They are a severe problem, which hinders the achievement of the goal to slow down climate change. Originated in the economic literature, macro level rebound effects refer to the gap between an aggregated economy’s potential and actual overall emission savings (Gillingham et al., 2016; Santarius et al., 2018)^1^, while micro rebound effects refer to the gap between potential and actual greenhouse gas emission savings of individual consumers or households (e.g., Sorrell, 2007; Chitnis et al., 2013). In this article, we focus explicitly on rebound effects on the single consumer level. These rebounds occur when consumers’ realized greenhouse gas emission savings caused by behaviors that might be beneficial to the environment, such as saving of conventional energy (e.g., through technical improvements) or the abandonment of harmful consumption (e.g., meat consumption), are lower than their potential greenhouse gas emission savings because the savings are partially or fully offset or even overcompensated by behavioral adjustments that are relatively detrimental to the environment (e.g., Reimers et al., 2021).^2^

Previous studies have intensively researched the economic mechanisms that lead to rebound effects on the consumer level (e.g., Sorrell et al., 2009, 2018; Sorrell, 2012; Azevedo, 2014; Font Vivanco et al., 2018). These mechanisms are based on income and substitution effects (Chitnis and Sorrell, 2015; Gillingham et al., 2016). Moral-psychological mechanisms can, however, also stimulate rebound effects on the consumer level. However, as outlined by the few exceptions of conceptual papers that started discussing these effects (e.g., Dütschke et al., 2018, 2021; Sorrell et al., 2020; Reimers et al., 2021), these effects have thus far been largely ignored in empirical research. The concept of moral licensing (Merritt et al., 2010; Mullen and Monin, 2016) has recently emerged as a promising theoretical foundation for the moral-psychological mechanisms. Calls become louder to apply this theory to shed light on the relationship between consumers’ initial moral actions and their subsequent behavioral adjustments that attenuate the potential greenhouse gas emission savings (Dütschke et al., 2018; Sorrell et al., 2020; Reimers et al., 2021). We claim that moral licensing theory can also help explain inconsistent behavioral consumption patterns across different product categories and consumption contexts. Applying moral-psychological mechanisms to explain rebound effects on the consumer level, therefore, bears the potential to account for a wide array of effects, which are beyond the realm of economic explanations. This study, therefore, develops the conceptual and theoretical foundation to stimulate research in this promising future research area.

Research on rebound effects on the consumer level distinguishes between direct and indirect rebound effects (e.g., Sorrell, 2007; Chitnis et al., 2014; Dütschke et al., 2018). Direct rebound effects describe that consumers’ actual greenhouse gas emissions in one product category increase after they have performed certain greenhouse gas emission reducing behavior in that same category, while indirect rebound effects describe that consumers increase greenhouse gas emissions in another product category (Chitnis et al., 2014). In this article, we focus on the individual’s subjective view, claiming that the borders across product categories are more blurred from a consumer-centered perspective. We expand the literature on categorization, which confirms that consumers apply individual perceptions of categories and that they even create categories to group objects for their purposes (e.g., Loken, 2006; Loken et al., 2008). We propose that the same processes occur for consumption contexts (e.g., private vs. work contexts). These individual perceptions of product categories and consumption contexts call for research on multifaceted indirect rebound effects. To disentangle these complex mechanisms, we suggest a clear multidimensional conceptualization of rebound effects on the consumer level.

This conceptual paper makes several contributions to the literature: First, we introduce a well-structured conceptualization of rebound effects on the consumer level. We define three dimensions: mechanism of rebounds (economic or moral), product category (same or different), and consumption context (same or different). We combine these three dimensions to develop a conceptualization termed the “rebound cube,” which categorizes different types of direct and indirect rebound effects on the individual level. Second, based on this new conceptualization and integrating assumptions from the theory of moral licensing, the theory of categorization, and the construal level theory, we outline promising directions for future empirical research.

The remainder of the paper is organized as follows. First, we provide the theoretical basis of our conceptualization. Next, we introduce our novel model of rebound effects on the consumer level. Finally, we outline implications for future research.

## Theoretical background

In this chapter, we explain the theoretical and conceptual foundations that are essential to our conceptualization and our later discussion. First, we disentangle basic concepts such as rebound, spillover effects, and moral licensing. Subsequently, we introduce the concept of moral licensing (e.g., Miller and Effron, 2010; Merritt et al., 2010; Mullen and Monin, 2016), which serves as a theoretical background of moral-psychological mechanisms of rebound effects on the consumer level, which will be explained more deeply in section “Economic or moral-psychological mechanisms.” Afterward, we portray the theory of categorization (e.g., Barsalou, 1983; Loken et al., 2008), which helps explain consumers’ subjective categorization processes of product categories and consumption contexts. We will refer to the theory of categorization in sections “Same or different product category” and “Same or different consumption context” to discuss the role of product categories and consumption context in explaining direct and indirect rebound effects. Lastly, we introduce the construal level theory (Trope and Liberman, 2003, 2010), which we will use in chapter. Directions for future research on rebound effects from a consumer-centered perspective to link the insights from the concept of moral licensing and the categorization theory to propose that indirect rebound effects occur frequently.

### Definition of rebound effects and related concepts

The literature does not consistently distinguish between the concepts of rebound effects, negative spillover effects, and moral licensing. Instead, these concepts are often described as overlapping, or they are used interchangeably (e.g., Truelove et al., 2014; Nash et al., 2017; Nilsson et al., 2017; Galizzi and Whitmarsh, 2019). Notably, relevant distinctions exist (Reimers et al., 2021).

To avoid confusion, we define rebound effects as the quantified gap between consumers’ actual greenhouse gas emission savings and the emission savings that they could potentially realize using energy saving alternatives or through more sufficient consumption (Sorrell, 2007; Guerra and Sancho, 2010; Thomas and Azevedo, 2013; Chitnis et al., 2014; Dütschke et al., 2018). We use the term “rebound effect” without any assumptions about the underlying (economic or moral-psychological) mechanisms that may cause rebound effects.

Behavioral spillover effects describe that a consumer’s initial behavior positively or negatively influences the probability of their subsequent behavior (Nilsson et al., 2017). In the context of morally laden behavior (i.e., pro-environmental behavior) a behavioral spillover effect indicates that one morally laden behavior influences the occurrence of another morally laden behavior. Positive spillover effects occur if the subsequent behavior is also morally laden. Negative spillover effects refer to immoral subsequent behavior (Nilsson et al., 2017; Galizzi and Whitmarsh, 2019). Positive spillovers are therefore consistent behavioral patterns from a moral standpoint, while negative spillovers are inconsistent behavioral patterns (Thøgersen, 1999; Juhl et al., 2017; Galizzi and Whitmarsh, 2019; Mai et al., 2021). Negative behavioral spillover effects can cause rebound effects. Again, we use the term “spillover effect” without implying any (economic or moral-psychological) mechanism that explains the reason for the negative relationship between two (e.g., morally laden) behaviors.

We refer to moral licensing to describe the underlying moral-psychological mechanisms of negative spillover effects, which can ultimately lead to rebound effects (Nilsson et al., 2017; Dütschke et al., 2018; Reimers et al., 2021). Other mechanisms, such as social-psychological or sociological mechanisms, where environmentally friendly behavior is influenced by standards and norms of the social environment may also lead to negative spillover effects (see Reimers et al., 2021). In this paper, we focus on moral-psychological mechanisms of rebound effects. To summarize, we use the term “rebound effects” to focus on the outcome, “spillover” to describe behavioral reactions, and “moral licensing” to describe the moral-psychological mechanisms.

### Moral licensing

The concept of moral licensing describes the phenomenon that “past good deeds can liberate individuals to engage in behaviors that are immoral, unethical, or otherwise problematic, behaviors that they would otherwise avoid for fear of feeling or appearing immoral” (Merritt et al., 2010, p. 344). Within the extensive research on moral licensing (for a meta-analytical overview, see Blanken et al., 2015), there is a consensus on two possible processes that can explain moral licensing: *moral credits* and *moral credentials* (Miller and Effron, 2010; Mullen and Monin, 2016).

According to the moral credits view, morally laden deeds, i.e., actions that can be considered morally good, create moral credits, which can be regarded as a moral currency. Individuals can, metaphorically speaking, deposit and withdraw these moral credits from their moral bank account (Miller and Effron, 2010). After having performed a morally laden deed, they can use their moral savings at a later point in time to offset a less moral deed. Notably, individuals will still interpret the immoral behavior as a moral transgression. They can, however, equalize the transgression by a former good deed. This view thus implies a fluctuation of the moral self-concept through subsequent moral and immoral behaviors (e.g., Sachdeva et al., 2009; Zhong et al., 2009; Miller and Effron, 2010). The moral credentials view assumes that individuals who have performed moral actions in the past change the way how they perceive their behavior in such a way that they will not fear being evaluated as questionable by themselves or observers—even if they actually are (Monin and Miller, 2001; Merritt et al., 2012).^3^ The moral credentials view therefore proposes that specific deeds do not affect the moral self-concept (Effron and Monin, 2010; Mullen and Monin, 2016).

Previous studies have confirmed that moral licensing shapes behavior in various domains, including ethical behavior of group leaders in a working context (Wang and Chan, 2019), consumer decisions linked to morality, e.g., buying self-indulgent products or the amount of donations for charitable purposes (e.g., Khan and Dhar, 2006; Chang and Chen, 2019), food choices (e.g., Wilcox et al., 2009), company–NGO collaborations (Schlegelmilch and Simbrunner, 2019), and climate/environmental behavior (e.g., Sachdeva et al., 2009; Mazar and Zhong, 2010; Meijers et al., 2015). A number of scholars have already transferred the theory to the context of rebound effects, where the immoral action is usually accompanied by relatively high greenhouse gas emissions (e.g., Panzone et al., 2012; Harding and Rapson, 2019). Several moderators have been suggested to explain whether initial moral behaviors lead to consistent behavioral patterns or to licensing, including the level of mental construal (Mullen and Monin, 2016; Lasarov and Hoffmann, 2020). We use the concept of moral licensing which serves as a theoretical background of moral-psychological mechanisms of rebound effects on the consumer level, which we explain more deeply in section “Economic or moral-psychological mechanisms.”

### Theory of categorization

Categorization describes the process when individuals use categorical representations “to assign a particular product or service to a consumer category, so that they can understand and draw inferences about it” (Loken et al., 2008, p. 133). Consumers use categories to group related objects (Loken et al., 2008). Early research on categorization theory suggests that consumers can make use of common taxonomic categories, such as bikes and cars, but they may also create categories that match their personal goals (Barsalou, 1983, 1985). For example, when a consumer plans a vacation, he or she may categorize vacation options either along the categories types of transportation or places to go (Barsalou, 1985, p. 632).

Goal-derived categories allow cross-categorization of objects. The goal-derived categories can consist of several previously established categories or specific subgroups of categories (Barsalou, 1983, 1985), and they can include categories that do not necessarily share the same characteristics (Ratneshwar et al., 2001). For example, the places to go may include beaches, pyramids, four-star hotels, and Australia. *Ad hoc* categories are goal-derived categories that individuals actively construct in a particular situation to achieve novel goals, for example, finding an alternative mobility concept to go to work when the car is broken. *Ad hoc* categories are therefore not based on well-established category representations in memory like common taxonomic categories or goal-derived categories that are more frequently used (Barsalou, 1983, 1985, 1991).

Research on consumer categories concludes that consumers’ interaction with various products and with a steadily changing environment requires categorical representations to be stable and flexible (Loken, 2006; Loken et al., 2008). According to Loken (2006, p. 458) “consumers need to have stable representations of objects and events in memory that can be used for interpreting” and “evaluating object and also require flexibility and the ability to adapt to changes in the environment.” Accordingly, to draw inferences about objects, individuals use representations of product categories that are stable over time and across different product categories (Loken et al., 2008). Still, depending on the usage context and personal goals, category boundaries and the assignment of objects to categories be also malleable and flexible (Ratneshwar and Shocker, 1991). We will refer to the theory of categorization to discuss how consumers can individually define the product categories (see section “Same or different product category”) and consumption contexts (see section “Same or different consumption context”) in the context of pro-environmental behavior that may lead to rebound effects.

### Construal level theory

The level of construal has been suggested as one moral licensing moderator (Mullen and Monin, 2016; Lasarov and Hoffmann, 2020). We will now briefly introduce the construal level theory (CLT; Trope and Liberman, 2003, 2010). While we need the concept of moral licensing and the categorization theory to develop the rebound cube, we will use the CLT to derive further propositions. Hence, in section “Directions for future research on rebound effects from a consumer-centered perspective,” we will combine the CLT with the concept of moral licensing and the categorization theory to explain how consumers can intentionally define the construal level to strategically define product categories and consumption contexts across which moral-psychological rebound effects occur.

The construal level theory (Trope and Liberman, 2003, 2010) proposes a relationship between a consumer’s psychological distance to an object or event and the extent to which the consumer’s thinking about the object or event is abstract or concrete. The level of construal refers to the level of abstraction (low vs. high abstraction level) and is thus related to how narrow (concrete) or broad (abstract) objects are categorized (Liberman et al., 2002). Psychological distance is defined as the “subjective experience that something is close or far away from the self, here, and now” (Trope and Liberman, 2010, p. 1). For example, participants in a study by Liberman et al. (2002) were asked to imagine different leisure activities (e.g., camping trip; moving out) that would take place in a year (distant) or next weekend (proximate). They were then asked to group different objects (e.g., brush, tent, matches; desk, VCR, pets) into the same set of objects. Individuals who were asked to imagine the activity in 1 year created fewer and thus broader categories (Liberman et al., 2002). According to the construal level theory, consumers can only directly experience what is in the immediate environment. Psychological distances emerge in temporal, spatial, social, and hypothetical dimensions. Regarding the interplay between psychological (temporal distance) and the level of construal and illustrated in the study by Liberman et al. (2002), for example, CLT suggests that individuals are more inclined to classify objects into abstract categories for situations in the distant future than for situations in the proximate future.

Whereas low-level construals are more related to subgoals and explain how an action is supposed to be done, high-level construals are more general and thus explain the reasons or superordinate goal for a performed action (Mullen and Monin, 2016). Accordingly, closer distances lead to more concrete thoughts on the object (low-level construal), thus focusing on the subordinate, concrete level including detailed information about an object. Higher distances lead to more abstract thinking about the object (high-level construal), focusing on the superordinate, abstract level, and removing information that is unnecessary according to the goal that the mental representation is chosen for (Trope and Liberman, 2010).

## A new conceptual foundation for rebound effects on the consumer level

We now introduce a new conceptual foundation for direct and indirect rebound effects on the consumer level, which is structured along three continuous dimensions, with the first ranging from economic to moral-psychological mechanisms of rebounds, the second ranging from the same to a different product category, and the third ranging from the same to different consumption context. For the sake of simplicity, we focus in this article on the anchors of these dimensions (economic vs. moral-psychological mechanisms, same vs. different product category, same vs. different consumption context). However, all three dimensions are continuous (from economic via mixed to moral-psychological mechanisms, from the same product category via decreasing similarity to completely unrelated product categories, from the perception of a highly similar consumption context to very distant consumption contexts). Moreover, we explicitly distinguish between the dimensions of product category and consumption context as both of these external factors, object (= product category) and the setting (= consumption context) can influence consumption behavior (Belk, 1975). Remarkably, we will later show that the manner how consumers categorize product categories and consumption contexts follow the same logic and we will use the theory of categorization to explain the perceived similarities for both dimensions (Loken et al., 2008). As we later demonstrate consumers could even (strategically) define product categories and consumption contexts as similar or different. Therefore, the subjective perspective of consumers can create a wide spectrum of indirect rebound effects via moral licensing.

### Economic or moral-psychological mechanisms

Rebound effects can be stimulated by *economic mechanisms* that are based on income effects and/or substitution effects (Chitnis and Sorrell, 2015; Gillingham et al., 2016). When consumers save greenhouse gas emissions by switching to a more energy-efficient consumption alternative, they often also save money. Income effects occur when the cost savings due to the lower energy consumption are re-invested in consumption. If this consumption is associated with additional greenhouse gas emissions, a rebound effect occurs (Chitnis and Sorrell, 2015).

Based on the concept of moral licensing, rebound effects can also be stimulated by *moral-psychological mechanisms* (e.g., Santarius et al., 2016; Dütschke et al., 2018, 2021; Santarius and Soland, 2018; Sorrell et al., 2020; Reimers et al., 2021). Consumers might believe that they have done something good for the environment if they switch to a consumption alternative involving less greenhouse gas emissions. If consumers, for example, switch to an energy-efficient e-bike vs. a car with a combustion engine, they may gain moral credits that liberate them to act less sustainably in a subsequent act of consumption.

Economic and moral-psychological mechanisms may occur independently of each other and they may also overlap. The simultaneous occurrence of both mechanisms may increase the rebound effects, compared to rebounds that are stimulated by a single (moral-psychological or economic) mechanism. However, even when a sustainable and greenhouse gas emission saving action is not associated with financial savings (e.g., buying more expensive organic food instead of conventional food), moral-psychological rebound effects may occur.

### Same or different product category

The term product relates to all tangible products and intangible products (services). The purchase of a product or the use of a service may involve monetary or non-monetary (e.g., time) expenditures. A product category refers to a group of products that share similar benefits.

Rebound effects can occur within the *same product category* (direct rebound effects) or between *different product categories* (indirect rebound effects; Chitnis et al., 2014). The study of Tiefenbeck et al. (2013) provides an example of negative spillover effects across different product categories. The authors found that providing consumers with feedback about the decrease of their water consumption causes an increase of consumption in another product category, namely electricity usage.

However, research highlights that consumers individually define product categories (Alba and Hutchinson, 1987; Cohen and Basu, 1987; Loken et al., 2008; Chowdhury and Feisal, 2020). They may even individually define specific product categories to serve a particular goal (e.g., Loken, 2006; Loken et al., 2008). Consequently, consumers may have their own perception about whether two products belong to the same product category or to different categories. For example, one consumer may ascribe using an e-bike and using an airplane for a holiday trip as two different categories (riding vs. flying), whereas another person may ascribe both to the same category (mobility concept). Along the same lines, one person may consider that meat-based dishes and plant-based dishes belong to the same category (food), whereas another person ascribes them to different categories (meat vs. vegetarian food). So far, the literature is ambiguous about whether moral licensing or spillover effects are more likely to occur in the same or different domains of actions. The meta-analytic review by Blanken et al. (2015) could not find any difference in the size of moral licensing effects in the same and different domain of actions. However, this research does not specifically focus moral licensing in the context of environmentally friendly behavior, but rather in different fields of action (e.g., donations, heating, volunteering). Thøgersen and Crompton (2009) conclude that positive spillover effects in the context of pro-environmental behavior become less likely for dissimilar behaviors. In line with that, the meta-analysis by Maki et al. (2019) found positive spillover to be less likely and negative spillover effects to be more likely for low similarity of two pro-environmental behaviors.

Particularly for the moral-psychological mechanisms of rebound effects, it is relevant to understand whether consumers ascribe two products to the same or to different categories, as the moral-psychological processes that enable subsequent licensing behavior may differ for within-category and cross-category constellations. For example, it may be subjectively easier to justify a cross-domain effect than a within-domain effect, because two actions in the same domain are more comparable and immoral deeds can therefore completely neutralize the former moral deeds in the same category. The morality of two purchases or usages in different product categories is more difficult to compare, which allows for more subjective interpretation and flexibility, which may ultimately lead to more intensive processes of moral licensing.

### Same or different consumption context

While there is no general accepted definition of consumption contexts, we refer to the literature on spillover effects to define consumption context as the setting in which a product or service is used (e.g., during holiday, work or, leisure time; Nilsson et al., 2017). Past research has demonstrated that contextual and situational factors influence consumer behavior (Belk, 1975) and moral behavior (Jones and Kavanagh, 1996; Tsang, 2002). In particular, these factors influence sustainable consumption behavior (Schultz et al., 1995; Simpson and Radford, 2014; Steg et al., 2014).

Rebound effects can occur in the same consumption context. For example, after a consumer has potentially saved greenhouse gas emissions in a certain consumption context he or she may later increase greenhouse gas emissions in the same. For example, consumers using the train instead of a private car (product category) for a private holiday trip (private consumption context) may later use the plane instead of a private car (product category) in a later private holiday trip (private consumption context).

A different consumption context means that a consumer’s potential greenhouse gas emission savings in one consumption context are attenuated by greenhouse gas emissions produced in another consumption context. For example, consumers using the train (product category) for a business trip (work context) may later use the plane (product category) for a private holiday trip more often (private context) which may be stimulated by moral credits gained.

Previous studies on behavioral spillover effects have already considered the role of consumption contexts with regard to two subsequent climate-relevant actions (e.g., Barr et al., 2010; Littleford et al., 2014). As stated in section “Same or different product category,” past research suggests that positive spillover effects are more likely for highly similar pro-environmental behaviors and negative spillover effects are more likely in less similar pro environmental behaviors (Thøgersen and Crompton, 2009; Maki et al., 2019). This literature however, mainly focuses on classes of pro-environmental behaviors (or product categories) and therefore do not take into account the consumption contexts. Even though previous studies have highlighted the importance to consider different contexts when analyzing behavioral spillover effects (e.g., Nilsson et al., 2017), the role of consumption contexts as neither been sufficiently discussed nor empirically tested with regard to rebound effects.

Referring to the theory of categorization (Loken et al., 2008), individuals may categorize their everyday consumption behavior into different consumption contexts, i.e., the context in which a product or service is used. The consumer’s perception determines the boundaries between different consumption contexts and consumers may even define these boundaries in favor of their personal goals. For example, one consumer may assign the two actions of riding a bike in her or his leisure time and riding a bike for business trips to the same consumption context (e.g., any consumption context). Another consumer may allocate the two actions to different consumption contexts (private context vs. work context). Again, this distinction is particularly relevant for explaining whether consumers use an initial act as a license for subsequent actions that lead to rebound effects.

### Literature about rebound effects on the consumer level

Empirical research about rebound effects on the consumer level has thus far mainly focused on economic rebound mechanisms, including direct and indirect rebound effects (for an overview, see, e.g., Sorrell et al., 2009, 2018; Sorrell, 2012; Azevedo, 2014; Font Vivanco et al., 2018). These studies analyze data based on consumer expenditure surveys or aggregated consumer demand statistics (Reimers et al., 2021), and thus they do not include specific information on single consumption contexts. Also, there is a number of empirical literature on spillover effects in the context of pro-environmental or climate-relevant behavior, mainly not considering the underlying psychological mechanisms (for a review see Maki et al., 2019; Geiger et al., 2021). Empirical studies on moral licensing in the context of pro-environmental or climate-relevant behavior are scarce (Phipps et al., 2013; Dütschke et al., 2018, 2021; Reimers et al., 2021). The literature on moral licensing offers fewer studies where the both sequential (im)moral actions are connected to consumption decisions (e.g., Catlin and Wang, 2013; Meijers et al., 2019; Burger et al., 2022) and thus allow to differentiate between same and different categories and contexts (for an extensive literature review, see Sorrell et al., 2020; Reimers et al., 2021). However, different consumption contexts have thus far rather been considered in the literature on behavioral spillover. With our novel conceptualization, we consider for the first time simultaneously different mechanisms, product categories and consumption contexts in rebound research. Table 1 illustrates how previous research on different research streams on economic rebound effects, moral licensing and spillover effects can exemplarily be classified along the three dimensions of the rebound cube.

**TABLE 1:** Overview of recent research that contributes to rebound cube.

Research stream	Article	Rebound cube dimension			Main findings that contribute to the rebound cube
		Mechanism	Category	Context	
	Davis, 2008	⚬	=	nc	Direct economic rebound effects from the usage of highly efficient washing machines: Households increased clothes washing after receiving a new washer.
***Economic Rebound Effects***	Buhl and Acosta, 2016	⚬	≠	nc	Resource sufficiency based indirect rebound effects in the domains of food, housing, and transport, from lowering room temperature, avoiding short car trips, and reducing food waste.
	Chen et al., 2019	⚬	= , ≠	nc	Direct and indirect rebound effects from transport: Efficiency improvements for urban passenger transport lead to an increased demand for all commodities.

	Catlin and Wang, 2013	●	=	=	Moral licensing effects within the same product category and consumption context: The possibility to use a recycling bin subsequently increased the amount of paper and paper towel consumption.
***Moral Licensing***	Meijers et al., 2019	●	≠	=	Moral licensing effects across different product categories in the same consumption context for people with low environmental identity: For example, the purchase of “green” sneakers lower behavioral intentions to perform different environmental activities (e.g., willingness to pay more for electricity to support clean air).
	Noblet and McCoy, 2018	●	≠	=	Moral licensing effects across different consumption categories in the same consumption context: People who stated that they formerly had performed sustainable activities were less likely to support a public policy investment in energy efficiency/renewable energy. These effects are mediated by an internal environmental motivation.

	Tiefenbeck et al., 2013	●	= , ≠	=	Negative spillover cross-category effects caused by environmental feedback in the same context: Individuals decreased water consumption but at the same time increased electricity usage.
***Behavioral Spillover Effects***	Barr et al., 2010	nc	= , ≠	≠	Different consumption contexts and lifestyle groups need to be considered when analyzing pro-environmental consumption behavior. Certain people behave less environmental friendly when on holiday than when at home.
	Littleford et al., 2014	nc	= , ≠	=, ≠	The consumption context (home vs. work) is an important factor influencing energy use behavior. However, this research could not support spillover effectsacross contexts.

Mechanism: ●, moral-psychological; ⚬, economic; Category: =, same; ≠, different; Context: =, same; ≠, different; nc, not considered. We selected examples of studies from previous research that can be clearly categorized according to the rebound-cubes dimensions. For a literature review, please consult section “Literature about rebound effects on the consumer level”.

### Rebound cube

**FIGURE 1:**
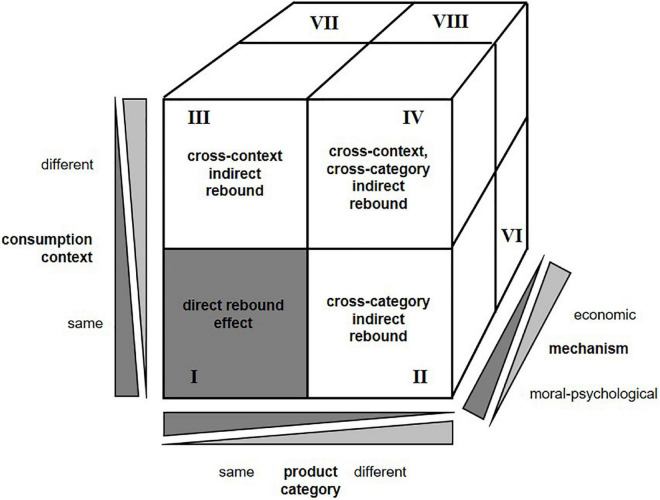
Rebound cube.

The three bipolar dimensions of mechanisms, product categories, and consumption contexts span up a three-dimensional model of rebound effects, which we term the rebound cube (depicted in Figure 1). This model builds the bases to describe different processes that lead to direct and indirect rebound effects on the consumer level.

This conceptualization outlines several novel and relevant aspects. First, there is not just a simple distinction between direct and indirect rebounds: Based on the combination of the three dimensions, we identify eight different types of rebound effects on the consumer level. Notably, while we use the anchors of the three dimensions to distinguish eight parts of the cube for the sake of simplicity, all dimensions are continuous. This is visualized in the figure by increasing and decreasing gray triangles. Rebound effects can be based on moral-psychological and/or economic mechanisms, and occur as direct, cross-category, cross-context, or cross-category and cross-context rebound effects. Second, these types of rebound effects are less well defined than initially thought. Given that consumers can individually and even strategically define product categories and consumption contexts, studies on rebound effects on the consumer level need to consider the variations of the individual perceptions. In the following, we will suggest directions for future research to disentangle these processes.

To systematically describe the eight different types of rebound effects, we refer to the illustrative introductory example. Imagine that the company iReliefs intends to involve its employees more in the company’s sustainability strategy. Sales representatives are asked to use their conventional bikes or e-bikes to travel to appointments at different locations (e.g., supermarkets, train stations, newspaper shops). To replace private car rides, both Oeconomica and Moralis bought an e-bike from their first salary, thereby setting the stage to save greenhouse gas emissions. However, instead of realizing these emission savings, both adjust their consumption behaviors. While economic mechanisms explain Oeconomica’s behavioral adjustments, moral-psychological mechanisms explain Moralis’ behavioral adjustments. The effects could potentially occur in the same product category (mobility concept) or in different product categories (e.g., food), and in the same consumption context (private setting) or in different consumption contexts (work setting). Table 2 systematically illustrates all eight potential rebound effects based on the new conceptualization. A moral-based direct rebound effect (rebound-type 1), for example, may occur, because Moralis had formerly tried to reduce the usage of the car with combustion engines by using a conventional bike (mobility concept) for shopping trips in her leisure time (private setting). However, she now spends the moral credits to use the e-bike (mobility concept) for every possible shopping trip (private setting).

**TABLE 2:** Examples of rebounds according to the rebound cube.

	Rebound-type	Scenario	Configuration			Example
				
No.	Name	Mechanism	Mechanism	Category	Context	Category and context of rebound
I	Moral-based direct rebound		●	**=**	**=**	While she had formerly tried to reduce the usage of the car with combustion engines (e.g., riding a conventional instead of using the car for shopping trips in her leisure time), she now spends the moral credits to use the e-bike for every possible shopping trip.
II	Moral-based cross-context indirect rebound		●	**=**	**≠**	While she had formerly used her conventional bike for all routes at work, she spends the moral credits that she had earned in her leisure time for using the new e-bike also for business trips at work.
III	Moral-based cross-category indirect rebound	By switching to the more energy-efficient e-bike compared to a car with a combustion engine *Moralis* earns moral credits as she does something good for the environment. She can spend the moral credits in the same or different category and context.	●	**≠**	**=**	Now that she uses a more energy-efficient mobility concept, she allows herself to consume less sustainably in different product category. She spends the moral credits for consuming meat dishes at home more often.
IV	Moral-based cross-category, cross-context indirect rebound		●	**≠**	**≠**	Now that she uses a more energy-efficient mobility concept for private purposes, she allows herself to consume less sustainably in a different product category at work. She spends the moral credits for choosing the meat dish over the vegetable dish in the canteen at work more often.

V	Economic-based direct rebound		⚬	**=**	**=**	While she had formerly tried to reduce the usage of the car with combustion engines (e.g., riding a conventional bike instead using the car for shopping trips), she now spends the additional financial resources to use e-bike for every possible shopping trip.
VI	Economic-based cross-context indirect rebound		⚬	**=**	**≠**	While she had formerly used her conventional bike for all routes at work, she spends the additional financial resources that she had earned in her leisure time for using the new e-bike also for business trips at work.
VII	Economic-based cross-category indirect rebound	By switching to the more energy-efficient e-bike compared to a car with a combustion engine *Oeconomica* earns additional financial resources, e.g., due to income effects. She can spend the financial resources in the same or different category and context.	⚬	**≠**	**=**	Now that she uses a more energy-efficient mobility concept, she saves money that she can spend in a different product category. She spends the additional financial resources for consuming meat dishes at home more often.
VIII	Economic-based cross-category, cross-context indirect rebound		⚬	**≠**	**≠**	Now that she uses a more energy-efficient mobility concept for private purposes, she saves money that she can spend in a different product category at work. She spends the additional financial resources for choosing the meat dish over the vegetable dish in the canteen at work more often.

Mechanism: ●, moral-psychological; ⚬, economic; Category: =, same; ≠, different; Context: =, same; ≠, different. Examples do not refer to different perceptions of categories and contexts.

## Directions for future research on rebound effects from a consumer-centered perspective

Relying on the rebound cube as a conceptual basis, we propose directions that hopefully stimulate future research on the moral psychology of rebound effects. As we will show in the following, the category and context effects are highly relevant for the moral-psychological mechanisms, as these effects open up a large spectrum of possible licensing effects and thus various indirect rebound effects from a consumer centered perspective. To disentangle these effects, we combine the key elements of moral licensing theory, the theory of categorization, and the construal level theory with the three dimensions of the rebound cube to suggest promising avenues for future research. We call for research on the different quantification of economic and moral “currencies” that explain rebound effects on the consumer level, on the different characteristics of economic and moral currencies, on the strategic categorization of products, and on the strategic categorization of consumption contexts.

We now introduce three research avenues each derived from the three dimensions of the rebound cube: mechanism of rebounds (economic or moral-psychological), product category (same or different), and consumption context (same or different).

### Research avenue on rebound-cubes dimension: Economic or moral-psychological mechanisms

**Avenue 1:** *While monetary induced (in)direct rebound effects are associated with real emission savings, (im)moral acts cannot equally be translated into emission savings.*

*Different quantification of economic and moral currencies*. Both economic and moral-psychological mechanisms of rebound effects are based on a “currency.” The economic currency is easy to quantify and the more money a consumer saves in one consumption action, the more he or she can potentially spend in another consumption action. For example, a consumer may save 50 USD monthly by switching from a car to an e-bike, and he or she can spend this money on holiday trips. Economic rebound effects based on income and substitution effects are therefore well researched (for an overview on direct and indirect rebound research, see, e.g., Sorrell et al., 2009, 2012; Azevedo, 2014; Font Vivanco et al., 2018; Sorrell et al., 2018).

The currency of morally laden behavior (moral credits) is fuzzier and harder to quantify. Past research shows that consumers link environmentally friendly behavior to morality (e.g., Mazar and Zhong, 2010). However, different consumers will evaluate the moral impact of the same morally laden behavior differently due to individual factors (e.g., involvement, knowledge) and contextual factors (e.g., private, public consumption). Even if consumers are informed about the greenhouse gas emissions caused by their behavior, they may not understand the implications of this measure (White et al., 2019).

Factors that affect the subjective evaluation include, for example, that consumers have an inadequate understanding of the magnitude of their resource consumption (Beal et al., 2013; Grinstein et al., 2018) or the magnitude of the environmental consequences of their consumption; therefore, they either overestimate or underestimate the extent to which different behaviors affect the environment (Attari et al., 2010). Furthermore, due to self-serving biases, consumers may overvalue the morality of their actions and undervalue the severity of their moral transgression. Consumers may thus also strategically overvalue a reduction in one category (e.g., using a bike instead of the car to travel to work once a month) to gain a higher license to justify subsequent higher greenhouse gas emissions in another category (e.g., choosing meat-based dishes in the canteen more often). For example, while Consumer A considers switching to an e-bike as a strong reduction of her carbon footprint, Consumer B does not foresee many merits for the environment.

Since previous research has quantified rebound effects based on economic units for products or services that can be clearly connected to particular measure, such as energy use or greenhouse gas emission (e.g., Chitnis and Sorrell, 2015; Gillingham et al., 2016), the starting point for future research on moral-psychological rebound effects should be a clearer understanding of subjective evaluations of moral deeds and the individual estimation of the environmental impact of moral deeds. As the literature on slacktivism shows, consumers may strategically apply even minor moral deeds to license larger transgressions (Skoric, 2012; Soyer et al., 2013). Recent research offers first approaches to connect sequential behaviors with a concrete measurable environmental consequence based on experimental studies. For example, a study by Tiefenbeck et al. (2013) shows that individuals who decreased water consumption at the same time increased electricity usage. These studies are therefore potentially suitable to quantify the magnitude of moral-psychological rebound effects based on a uniform measure, such as greenhouse gas emissions. Other studies capture consumers’ individual estimations of environmental consequences based on survey studies (Grinstein et al., 2018). Future research may use these approaches to combine moral credits with hard data on concrete measures in terms of environmental impacts, such as emission savings and at the same time captures individual’s perceptions of these consequences and the intrapersonal variations. In addition to more traditional moral licensing and spillover studies, research on technical solutions to measure consumers’ greenhouse gas emissions should also be considered. For example, carbon footprint tracking apps allow to capture and calculate the greenhouse gas emissions of consumption across different categories (Hoffmann et al., 2022). A pre-interview or survey of the app users would provide a solution to compare the concrete calculable emissions with consumers’ subjective perception of the emissions.

#### Different characteristics of economic and moral currencies

Economic transactions are linked to a fixed extrinsic value. Disregarding fluctuations, for example, when it comes to inflation or investment income, financial resources remain stable in the economic bank account until they are spent. Furthermore, consumers cannot invest the same saved money twice. By contrast, a single moral action can be used as moral licensing for multiple subsequent immoral actions. For example, consumers can rely on the same past moral deed on many different occasions for different product categories and consumption contexts. Besides, the empirical research showed that moral licensing mechanisms are not stable across a series of sequential behaviors; instead, they converge to an internal moral equilibrium in the long term (Barque-Duran et al., 2016). Referring to construal level theory, recent moral behavior evokes moral licensing, whereas temporally distant moral behavior leads to consistency (Conway and Peetz, 2012). These effects should be explored in future research on moral-psychological rebound effects. Multiple usage of the same unit of the moral currency is particularly possible across different product categories and consumption contexts.

As we will demonstrate below, the moral-psychological or economic mechanisms involved in consumer decision-making may affect in different ways how consumers (strategically) interpret whether products belong to the same or different product categories. We will also show how these mechanisms differently affect how consumers’ (strategically) interpret whether different consumption decisions are made in the same or in different consumption contexts.

### Research avenue on rebound-cubes dimension: Same or different product category

**Avenue 2:** *Consumers subjectively (and even strategically) interpret whether products belong to the same or to different product categories.*

*(Strategic) categorization of products based on moral-psychological and economic mechanisms*. Referring to section “Theory of categorization,” research on categorization suggests that individual goals and situational factors shape how individuals categorize objects and situations (Loken, 2006; Loken et al., 2008). Self-regulation mechanisms often involve goal hierarchies, i.e., decomposing a superordinate goal into many subgoals (Fishbach et al., 2006). For example, a consumer can have a superordinate goal to reduce the greenhouse gas emissions resulting from his or her consumption. The consumer might also decompose this superordinate goal into many more concrete subgoals, such as reducing greenhouse gas emissions in the household, mobility, etc. As stated in section “Construal level theory,” the level of construal has been suggested as a critical moderator of moral licensing mechanism (Mullen and Monin, 2016; Lasarov and Hoffmann, 2020). Accordingly, whether consumers focus on abstract goals or concrete goals influences if they feel morally licensed or not in a subsequent situation (Trope and Liberman, 2010; Mullen and Monin, 2016).

Focusing on achieving one concrete goal provides consumers with a moral license, whereas consumption in other goal relevant categories may be considered as substitutes (Fishbach et al., 2006). The question as to whether two consumption actions fall within the same product category or in different product categories can influence whether consumers interpret a certain action as a moral license. Past research on moral licensing indicates a strategic interconnection between sequential actions (Merritt et al., 2012; Lasarov and Hoffmann, 2020). For example, consumers may strategically rely on anticipated moral actions, to justify their present moral transgressions (Cascio and Plant, 2015; Lasarov and Hoffmann, 2020).

Consequently, the consumers may strategically categorize products into the same or different categories based on moral-psychological considerations. For example, a consumer who usually interprets riding a bike and driving a car as belonging to the same category (mobility), may strategically ascribe the two actions to different categories after reducing emissions of the category driving (e.g., the substitution of shopping trips by car with shopping trips by bike) to justify higher emissions of the other category (e.g., driving the e-bike more often).

Consumers may also strategically categorize product categories based on economic mechanisms. For example, the literature on mental accounting suggests that consumers use mental accounts to organize their finances for different consumption categories (e.g., Thaler, 1985, 1999). However, the strategic categorization of product categories purely based on economic mechanisms may occur due to other reasons that do not take into account the environmental consequences of the individual consumer decisions. For example, consumers may decide to construe different product categories in order to allow themselves additional spending in one product category due to monetary savings in another product category.

#### Strategic categorization of product category based on consumption contexts

In the context of rebound effects from a consumer-centered perspective, the strategic categorization of product categories may occur independently or dependent on the consumption contexts involved. Strategic product categorization may occur independently of the consumption context. In this case, it doesn’t matter whether consumers perceive two actions as belonging to the same context or whether they do not deliberately distinguish between different contexts at all.

The strategic product categorization may also interact with the individual consumption context. Since consumers can have perceptions of different consumption contexts, they can strategically categorize products depending on such contexts. For example, consumers who differentiate between work and private contexts, may strategically categorize the product categories related to their actions differently, as in the private context they are more free and flexible in their choices than in the work context. Consumers who may save greenhouse gas emissions in one product category due to the firms’ sustainability strategy (e.g., transportation type), can therefore either use the same product category in the private context to categorize their subsequent behavior (cross-context indirect rebound effect) or they may strategically categorize their behavior in the private context in more detailed categories, such as using the car, bus or train (cross-context, cross-category indirect rebound effects) to more easily justify the moral transgression.

Previous research on rebound effects has not considered these intrapersonal variations in the perception of product categories. Integrating this moral-psychological lens sets the stage to explore rebound effects that occur due to the strategic categorization of products. Notably, this more consumer-centered perspective implies that consumers can (strategically) reinterpret rebound effects that were previously considered direct rebounds, as indirect rebounds.

### Research avenue on rebound-cubes dimension: Same or different consumption context

**Avenue 3:** *Consumers subjectively (and even strategically) interpret whether different consumption choices are settled within the same or in different consumption contexts.*

*(Strategic) categorization of consumption contexts based on economic and moral-psychological mechanism*. Similar to the discussed (strategic) categorization of products, consumers might (strategically) decompose and categorize their consumption into different sub-contexts. To achieve the goal of reducing greenhouse gas emissions from personal consumption, consumers may derive different consumption contexts, such as reducing greenhouse gas emissions in the domain of mobility in the private, work, or social context. Consumers may strategically interpret whether different consumption choices are settled within the same or in different consumption contexts based on moral-psychological considerations. Different consumption contexts (work, leisure, sports, vacation, etc.) also determine consumers’ social environment (e.g., co-workers, family, friends) and thus also the consumers’ different social roles in these contexts. These roles in turn define different social norms and obligations for the consumers. Different social roles may therefore influence how and why consumers strategically shape different consumption contexts based on moral-psychological considerations. Consider a consumer who usually interprets riding an e-bike as belonging to the category mobility, independently of whether she uses the e-bike in a private context or in a work context. She may thus strategically assign riding an e-bike at work (work context) and riding it for private purposes (private context) to different contexts to justify higher emissions of riding at the workplace (e.g., using an e-bike instead of using a conventional bike) after reducing emissions in free time (e.g., using an e-bike instead of a car for shopping trips). For example, the because social role of a service-oriented worker requires faster and more reliable modes of transportation in the work context.

Consumers may also strategically categorize consumption contexts based on economic mechanisms. However, this strategic categorization of consumption contexts purely based on economic mechanisms may occur due to other reasons that do not take into account the environmental consequences of the individual consumer decisions. For example, individuals might decide to construe different consumption contexts in order to allow themselves additional spending in one consumption context due to monetary savings in another consumption context.

#### Strategic categorization of consumption contexts based on product categories

Whereas it is very unlikely, that consumers completely refrain from categorizing their daily objects into product categories, consumption contexts are more individually and flexibly construed. On one hand, consumers may construe strategically different consumption contexts independently from the product categories involved in their consumption decisions. For example, consumers may in general behave differently in work and private contexts independently from the specific pro-environmental actions, one reason being that they follow different norms and obligation in their social environment (e.g., family, leisure, sports, vacation). However, more likely, strategic categorization of consumption contexts may interact with the different product categories involved. Therefore, in the context of rebound effects from a consumer-centered perspective, the strategic categorization of consumption contexts may mainly occur based on former product considerations. For example, consumers who may save emissions in one product category may strategically construe different consumption context to justify transgression in the same product category (cross-context indirect rebound effect) or in another product category (cross-context, cross-category indirect rebound effects).

Similar to the discussion of product categories, adding a psychological perspective on consumption contexts can stimulate future research on moral-psychological rebound effects. For example, individuals could interpret even those rebound effects as indirect, which a third person would classify as direct.

## Conclusion

A deep understanding of rebound effects is essential to mitigate undesirable consequences of sustainable consumption and thus to promote the achievement of climate protection goals.

Rebound effects on the consumer level are thus far underconceptualized because past research has mainly considered the economic mechanisms that lead to rebound effects on the consumer level (see, e.g., Sorrell et al., 2009, 2018; Sorrell, 2012; Azevedo, 2014; Font Vivanco et al., 2018). The moral-psychological mechanisms that lead to rebound effects have, however, thus far not been sufficiently discussed in rebound research (e.g., Dütschke et al., 2018, 2021; Sorrell et al., 2020; Reimers et al., 2021). This research further refines the conceptualization of the moral component as an explanatory factor for rebound effects and advances knowledge on rebound effects on the consumer level by proposing a novel conceptualizing including three dimensions: the mechanism (economic vs. moral-psychological mechanisms of rebounds), the product category (same product category vs. different product categories), and the consumption context (same vs. different context). Whereas previous research simply distinguishes between direct and indirect effects, our conceptualization shows that indirect rebound effects are more diverse than previously suggested: They can be economic based and/or moral-psychological based, and could potentially occur in the same category or in different categories, and in the same context or in different contexts. Based on this novel conceptualization, we take a more consumer-centered perspective to show that the category and context effects are of particular relevance for the moral-psychological mechanisms, as they open up wide-ranging possible licensing effects. Our research thus contributes to a deeper understanding of indirect rebound effects, which have been less considered than direct rebound effects in the literature (Reimers et al., 2021).

We also specified numerous research areas that are presently underdeveloped. More research is needed to expand our understanding of subjective evaluations of moral deeds, and the differences in the characteristics of economic and moral currencies that explain rebound effects on the consumer level. Furthermore, we showed that future studies should take into account consumers’ (strategic) categorization of products and consumption contexts. This more consumer-centered perspective implies that rebound effects previously considered direct rebounds can also be interpreted as indirect rebounds. With regard to measures against rebound effects, our conceptualization can also offer several new insights. Research, practitioners, and policy makers should consider this novel understanding of indirect rebounds to develop effective intervention strategies.

## Author contributions

HR, WL, and SH: conceptualization and writing—review and editing and original draft preparation. HR and SH: visualization and project administration. SH: supervision. WL and SH: funding acquisition. All authors read and agreed to the published version of the manuscript.

## Footnotes

1 Macroeconomic rebound effects refer to analyzing computable general equilibrium effects, whereas microeconomic rebound effects refer to analyzing partial equilibrium effects. In this research, we focus on rebound effects on the level of the single consumers. These effects also have an impact on the whole economy. However, examining these impacts is not part of our research focus. Some research also explicitly considers meso-economic rebound effects as production-side and sector-level effects (e.g., Santarius, 2016; Santarius et al., 2016).

2 Past research has analyzed rebound effects with respect to different impact measures, such as energy, greenhouse gas emissions, or water consumption (for an overview see Reimers et al., 2021). In the context of greenhouse gas emission, some researchers also differentiate between direct and indirect emissions when analyzing indirect rebound effects (embodied emissions; e.g., Chitnis et al., 2014). We consider rebound effects in terms of greenhouse gas emissions in this research. Thereby, we do not specify whether direct or indirect emissions are involved, as our focus is not on quantifying rebound effects, but rather on explaining the underlying moral-psychological mechanisms. The conceptualization presented in this research is applicable to various impact measures. Formally, the rebound effect (R) is defined as R = (PES-AES)/PES = 1-AES/PES with PES being the potential emissions savings and AES being the actual emissions savings (Chitnis et al., 2013; Thomas and Azevedo, 2013; Reimers et al., 2021).

3 Recent research has coined the term social moral licensing to consider the influences of the social environment on moral licensing (Lasarov and Hoffmann, 2020). In this research, we focus on individual moral licensing.
